# Comments for Mertens et al. (2018), Glyphosate, a chelating agent—relevant for ecological risk assessment?

**DOI:** 10.1007/s11356-018-2506-0

**Published:** 2018-06-16

**Authors:** John T. Swarthout, Marian S. Bleeke, John L. Vicini

**Affiliations:** Monsanto Company, St. Louis, MO USA

To the Editor,

We read the publication by Mertens et al. (2018) and wish to address a critical piece of misinformation that may have been inadvertently perpetuated by the authors, namely, the widely held belief that glyphosate was patented as chelator. This myth results in further sophistry that this property enables glyphosate to sequester key nutrients in the phloem or in soil, thus reducing their bioavailability to plants.

The basis for the review (Mertens et al. 2018) was “to critically evaluate its [glyphosate’s] role in effects that are not readily explained by the inhibition of EPSPS,” specifically, to evaluate the “well-known” chelation properties of glyphosate. This faulty premise that glyphosate was patented as a chelator results in the authors’ supposition that glyphosate’s chelation properties present “potential additional environmental risk was never adequately considered” in the European Food Safety Authority’s regulatory risk assessment (EFSA 2015a, b).

The statement that “Glyphosate is also known as a potent chelator for minerals, a property that has been observed decades ago (Toy and Uhing 1964)” is misleading. The Stauffer Chemical Company patent is about phosphinic acids and their properties including as chelating agents and as chemical intermediates in the synthesis of phosphonic acids. Glyphosate is a phosphonic acid, not a phosphinic acid, and it is in this context that glyphosate is mentioned, as an example of a product that can be synthesized from a phosphinic acid. While glyphosate (in the patent named “glycine-methylenephosphonic acid”) is mentioned (page 7, example 14, paragraph 2), glyphosate is not the molecule being patented and there is no discussion of its chelating properties.

Monsanto did receive a patent for the herbicidal properties of glyphosate (Irani 1969), but the compound was discovered and synthesized independently, and was not acquired from Stauffer. Thus, glyphosate was never patented as a chelator by either Stauffer Chemical Company or Monsanto Company. Yet, somewhere this misinformation was first promulgated to be passed through numerous introductions to journal articles as if it is scientific evidence.

Undoubtedly, glyphosate can function as a chelator, along with many naturally occurring compounds such as citric acid and histidine. However, it is not a uniformly strong chelator of metals and is a much weaker chelator than hexadentate ligands such as nicotianamine, which has an important role in transport of micronutrients in plants (Clemens et al. 2013) and compounds such as EDTA which are used in micronutrient fertilizers. Furthermore, glyphosate does not significantly impact the bioavailability of micronutrients. Mertens et al. (2018) assert that the chelation properties of glyphosate could mediate the sequestration of key micronutrients in the phloem reducing their bioavailability to the plant. However, in Harris et al. (2012)—regrettably not included in this review—the authors calculated the relative speciation of glyphosate to metal ions (both micro- and macronutrients, including Fe^3+^, Fe^2+^, Cu^2+^, Zn^2+^, Mn^2+^, Ca^2+^, and Mg^2+^) in phloem, using the stability constants for the principal chelating agents in phloem including nicotianamine, histidine, cysteine, glutamic acid, and citrate as compared to glyphosate. Results showed that (1) in the absence of glyphosate, metal ions in phloem form complexes with naturally occurring chelators, with very little remaining as free aquo ions; (2) when glyphosate was included in the modeled calculations, it did not significantly change the speciation of metal ions; and (3) over 90% of the glyphosate in phloem was not bound to any metal. Harris et al. (2012) conclude “that glyphosate is largely unable to compete effectively with the biological chelating agents in phloem.”

Additionally, Duke et al. (2017) concluded, following a 2-year field trial of glyphosate use in glyphosate-resistant (GR) soybeans, that “Neither glyphosate nor the GR transgene affect the content of the minerals measured in leaves and seed, harvested seed amino acid composition, or yield of GR soybean. Furthermore, soils with a legacy of GR crops have no effects on these parameters in soybean.”

Finally, EFSA’s analysis (EFSA 2015b) considered “33 articles [that] draw conclusions on the interactions of glyphosate with micronutrients, nutrient uptake and metabolism. In particular, the ability of glyphosate to chelate micronutrients such as manganese, iron and phosphate and the resulting effects in plants are addressed.” In a summary of their evaluation (page 37, section 2.3.4), EFSA concluded that “the available scientific data suggest that the strong affinity of glyphosate and its metabolite AMPA to most soils prevents the uptake of these compounds by root systems of non-target plants.”

Perhaps the best evidence that glyphosate does not interfere with mineral uptake or utilization by plants comes from Roundup Ready crops. A single gene transcribes CP4 EPSPS, which is tolerant to glyphosate. If glyphosate was interfering with minerals through chelation, these plants would succumb to this effect, which clearly does not occur.

In summary, Mertens et al. (2018) assert that the evaluation of glyphosate was inadequate as agencies like EFSA did not sufficiently consider other properties of glyphosate including chelation. As outlined above, the basis for this assertion is not substantiated by the facts. This includes that glyphosate was never patented as a chelator and that the chelating properties of glyphosate do not support any hypothesis that glyphosate could play a significant role in limiting the bioavailability of micronutrients to plants. Finally, as editors and peer reviewers are burdened with increasing numbers of submissions, authors need to be diligent about reviewing and accurately relaying information from secondary sources and instead rely on primary references.

Sincerely,

John T. Swarthout, Ph.D.

Marian S. Bleeke, Ph.D.

John L. Vicini, Ph.D.
